# Innovative substance 2250 as a highly promising anti-neoplastic agent in malignant pancreatic carcinoma - in vitro and in vivo

**DOI:** 10.1186/s12885-017-3204-x

**Published:** 2017-03-24

**Authors:** M. Buchholz, B. Majchrzak-Stiller, S. Hahn, D. Vangala, R. W. Pfirrmann, W. Uhl, C. Braumann, A. M. Chromik

**Affiliations:** 1 Division of Molecular and Clinical Research, St. Josef-Hospital, Ruhr-University Bochum, Bochum, Germany; 2 Department of Molecular Gastrointestinal Oncology, Ruhr-University Bochum, Bochum, Germany; 3 Geistlich Pharma AG, Wolhusen, Switzerland; 4 Department of Internal Medicine, Knappschaftskrankenhaus, Ruhr-University Bochum, Bochum, Germany

**Keywords:** Taurolidine, Apoptosis, Chemotherapy, Cancer, Substance 2250

## Abstract

**Background:**

Former studies already revealed the anti-neoplastic properties of the anti-infective agent Taurolidine (TRD) against many tumor species in vitro and in vivo*.* Its anti-proliferative and cell death inducing capacity is largely due to its main derivative Taurultam (TRLT). In this study it could be demonstrated, that substance 2250 - a newly defined innovative structural analogue of TRLT - exhibits an anti-neoplastic effect on malignant pancreatic carcinoma in vitro and in vivo.

**Methods:**

The anti-neoplastic potential of substance 2250 as well as its mode of action was demonstrated in extensive in vitro analysis, followed by successful and effective in vivo testings, using xenograft models derived from established pancreatic cancer cell lines as well as patient derived tissue.

**Results:**

Our functional analysis regarding the role of oxidative stress (ROS) and caspase activated apoptosis showed, that ROS driven programmed cell death (PCD) is the major mechanisms induced by substance 2250 in pancreatic carcinoma. What is strongly relevant towards clinical practice is especially the observed inhibition of patient derived pancreatic cancer tumor growth in mice treated with this new substance in combination with its sharply higher metabolic stability.

**Conclusion:**

These encouraging results provide new therapeutical opportunities in pancreatic cancer treatment and build the basis for further functional analysis as well as first clinical studies for this promising agent.

## Background

Pancreatic ductal adenocarcinoma (PDAC) is the most lethal common cancer, usually diagnosed at an advanced stage when curative therapy is almost impossible [1]. It is the fourth most common cause of cancer-related death in Europe [2] and in the US [3]. The pancreatic adenocarcinoma typically has a poor prognosis with a five year relative survival rate of 4–5% [4]. In fact, incidence and mortality of PDAC are almost equal. Surgical resection is the only potentially curative therapy for pancreatic cancer. Because of the poor outcome associated with surgery only, the role of adjuvant therapies has been extensively evaluated. A series of studies revealed, that chemotherapy with gemcitabine or fluorouracil improves the overall survival of patients with pancreatic adenocarcinoma [5–8]. In the majority of cases a complete resection of the tumor is impossible. Therefore a palliative chemotherapy may be conducted to prolong survival and improve the quality of life. In these cases new combination therapies like FOLFIRINOX are investigated in clinical trials or are in use [7, 8] However, current chemotherapeutic agents are still disappointing due to their poor response and high toxicity. New and innovative agents have to be found to expand the therapeutic opportunities.

Taurolidine (TRD) is a substance derived from the aminosulfonacid taurine. Owing to its anti-inflammatory and its anti-microbial qualities, it has been clinically used primarily in peritonitis and catheter related blood stream infections [9]. 1997 Jacobi et al. could show for the first time, that TRD also applied an anti-proliferative and anti-neoplastic activity in vitro and in vivo [10]*.* This anti-neoplastic and apoptosis inducing effect could also be verified by other research groups [11] in a variety of cell lines derived from malignant tumors e.g. glioblastoma [12], melanoma [13], mesothelioma [14] and colon carcinoma [15]. Furthermore, latest reports about the systemic application of TRD in patients with gastric carcinoma and glioblastoma revealed promising results with almost absence of toxicity [16, 17].

The favorable safety profile of TRD renders this compound to a promising novel agent for the oncological care. However, the metabolic stability of TRD is limited due to its short half-life [18].

**Fig. 1:**
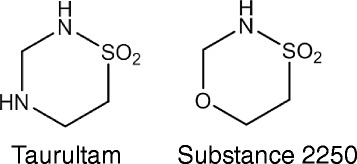
Molecular structure of substances TRLT and 2250. Substance 2250, 1.4.5-oxathiazan-dioxid-4.4, is an oxathiazine derivative like TRLT with a moleculare weight of 137.15 g/mol

This study was designed to analyze a novel compound related to Taurultam (TRLT), the main derivative of TRD. Substance 2250 is a structural analogue of TRLT and is an oxathiazine derivative (Fig. 1). The respective Sulfonamides are promising substances due to their antibacterial and anti-neoplastic activity. The 1.4.5-Oxathiazin derivatives, like the new substance 2250, are almost unexplored whereas 1.2.3-Oxathiazin derivatives are already identified, mainly as artificial sweeteners [19]. However, no representative studies are published analyzing the anti-neoplastic effects of the substance 2250 so far. The aim of this study was to investigate the anti-neoplastic activity on malignant pancreatic cancer in vitro and in vivo*.*

## Methods

### Cell lines and culture conditions

Six different human pancreatic cancer cell lines were used for our experiments: AsPC-1 (CLS Cell Lines Service, Eppenheim, Germany), BxPC-3 (ATCC – LGC Standards GmbH, Wesel, Germany), MiaPaca-2 (ATCC – LGC Standards GmbH, Wesel, Germany), Panc-1 (CLS Cell Lines Service, Eppenheim, Germany) and Panc-TuI (ATCC – LGC Standards GmbH, Wesel, Germany). Cells were passaged fewer than 6 months after receiving from the mentioned cell banks. Authentication was analyzed by STR analysis. MiaPaca-2, Panc-1 and Panc-TuI cells were cultured in Dulbecco’s Modified Eagle Medium (DMEM). The remaining cell lines (AsPC-1, BxPC-3) were maintained in RPMI 1640. All cultures were supplemented with the antibiotics penicillin (100 U/ml), streptomycin (100 U/ml) and 2 mM L-Glutamine. AsPC-1 cells were further supplemented with 1 mM Sodium Pyruvate. Cells were grown as monolayer and cultured in 25cm^2^ flasks at 37 °C and 5% CO_2_ in humidified atmosphere.

### Tissue

Different human pancreatic cancer tissues were used for the in vivo experiments:

Bo70, adenocarcinoma, UICC IIb.

Bo 80, adenocarcinoma, UICC IIb.

### Reagents

The 2250 and TRLT ultrapure powder (kindly provided by Geistlich Pharma AG, Wohlhusen, Switzerland) was dissolved in double distilled Water (ddH_2_O), sterile filtered, set to a physiological pH and freshly prepared once per week.

### MTT cytotoxicity assay

Cells were seeded to a density of 3.5 × 10^4^ cells/well in 96-well plates and incubated for 24 h to obtain a sub confluent monolayer. To examine the dose-response of 2250 and TRLT regarding its anti-neoplastic activity, cells were incubated with increasing concentrations (100, 200, 500, 1000, 1500, 2000 μmol/l) and ddH_2_O as control for 6, 12, 24 and 48 h. 4 h before the measurement 10 μl yellow MTT (3-(4,5-Dimethylthiazol-2-yl)-2,5-diphenyltetrazoliumbromid) reagent (5 mg/ml) was added to the test media. Yellow MTT is converted by viable cells into violet Formazan crystals. The test media was discarded and 50 μl DMSO (Dimethylsulfoxide) was applied. After an incubation time of 5–10 min the viability of cells could be analyzed by using a microplate absorbance reader by measuring the OD (optical density) (Tecan trading AG, Switzerland). The amount of violet Formazan is directly proportional to the amount of viable cells. The assay was performed in 4–6 independent experiments with consecutive passages.

### BrdU proliferation assay

Cells were seeded to a density of 4 × 10^4^ cells/well in 96-well plates and incubated for 24 h to obtain a sub confluent monolayer. To examine the dose-response of 2250 and TRLT regarding its anti-proliferative activity, cells were incubated with increasing concentrations of 2250 (100, 200, 500, 1000, 1500, 2000 μmol/l) and ddH_2_O as control for 6 h and submitted to BrdU proliferation assay (5-bromo-2-deoxyuridine)-ELISA (Roche Applied Science, Mannheim, Germany) according to the manufacturer’s instructions. Based on the incorporation of the thymidine analogue BrdU during DNA synthesis, the amount of synthesized DNA is detected using a microplate absorbance reader (Tecan trading AG, Switzerland). BrdU assays were performed with 8 replicates of three independent experiments with consecutive passages. The incubation time of 6 h has been shown to be appropriate for the BrdU proliferation assay in previous experiments.

### Flow Cytometry analysis

Cells were seeded to a density of 2 × 10^5^ cells/well in 6-well plates and incubated for 24 h to obtain a subconfluent monolayer. Different concentrations of 2250 (200, 500, 1000, 1500, 2000 μmol/l) and ddH_2_O as control were used for 24 h and 48 h before analyzed by FACS analysis. FACS analysis was performed in 4–6 independent experiments with 2–4 consecutive passages. Cells were fixed in 200 μl binding buffer (Bender MedSystems, Vienna, Austria). Subsequently, 5–10 μl Annexin V-FITC (BD Biosciences, Heidelberg, Germany) was added to the cell suspension and incubated for 15 min at room temperature in the dark. Thereafter, 10 μl Propidiumiodide (PI) (Bender MedSystems, Vienna, Austria) was added. Cells were analyzed immediately using a flow cytometer (FACS Calibur BD Biosciences, Heidelberg, Germany) for Annexin V-FITC (apoptotic) and PI (necrotic) binding. Dot plots and histograms were analyzed by CellQuest Pro software (BD Biosciences, Heidelberg, Germany).

### Functional mechanisms

To get insights into functional mechanisms of drug effects cells were additionally treated with the radical scavenger N-acetylcysteine NAC (5 mmol/l) (Sigma Aldrich, Munich, Germany) or the pan caspase inhibitor-zVad (2 μmol/l) (Enzo Life Sciences, Lörrach, Germany) before analysis by FACS or MTT standard procedure as already described. Concentrations of 500 and 1000 μmol/l 2250 with an incubation time of 24 h were used for co-incubation assays in all cell lines.

The direct impact of 2250 on the cellular level of reactive oxygen species (ROS) was analyzed using the Cellular ROS/Superoxide Detection Assay KIT (Abcam, Cambridge, UK) following the manufactures instructions.

### Analysis of the maximal tolerable dose

#### Acute toxicity

For determining the acute toxicity of substance 2250 in nude mice, mice (each group *n* = 8–10) were treated intraperitoneal (ip) with different concentrations (500, 1000, 1500, 2000 mg/kg*BW) once, followed by control of body weight and general vital function. A loss of 20% of body weight over 48 h was classified as toxic.

#### Chronic toxicity

To assess the chronic toxicity mice were treated with different concentrations of substance 2250 (500, 1000, 1500 mg/kg*BW) on alternating days for 3 weeks. Control of body weight and general vital function were measured as well. A loss of 20% of body weight over 48 h was classified as toxic.

### Analysis of metabolic half-life in blood

For determining the metabolic half-life of the new substance 2250 in the blood of nude mice, a colorimetric assay using NASH-Reagent and protein free serum was used. The serum level was analyzed 1 h and 25 h after treatment with the substance 2250 (500 mg/kg*BW).

### Animal studies

Five-week-old female NMRI Foxn1nu/Foxn1nu mice (Janvier, France) were acclimated into a 12-h light cycle-controlled environment 1 week before initiation of the study. The animals were allowed standard laboratory food and water ad libitum. Mice were anesthetized by inhalation of Isofluran. 5 × 10^6^ cells of different pancreatic adenocarcinoma cell lines (MiaPaca-2 and PancTU-I) or tumor tissue fragments were administered subcutaneously in the flank region. After implantation, the recipient mice were monitored for general health status and the presence of subcutaneous tumors. Tumor volume was determined by measuring tumor diameters (measurement of 2 perpendicular axes of tumor) using a caliper and calculated as.

$$ V=\frac{1}{2}\left( a{b}^2\right) $$,

(a = larger axe, b = smaller axe).

Following randomization (two groups, *n* = 10) the systemic influence of 2250 on tumor growth after intraperitoneal (ip) application were investigated. As the metabolic half-life of 2250 in nude mice was found to be 13.8 h, a dosing schedule of every second day was chosen for the in vivo experiments. Group1: substance 2250 500 mg/kg*BW on alternating days, group 2: control group Ringer’s solution 300 μl was applied ip on alternating days. The treatment was initiated when the tumor volume reached 200 mm^3^ and the tumor volume was measured every second day for 2–3 weeks. The experiment was terminated either after an application period of 3 weeks or when the tumor reached a volume of 1000 mm^3^.

### Statistics and calculations

Results of FACS-analysis (percentage of viable, apoptotic and necrotic cells) as well as results of MTT and BrdU assay (percentage of living/proliferating cells) are expressed as means ± SEM. Comparison between experimental groups with normal distribution was performed using one-way ANOVA followed by Tukey’s post-hoc test. For categorical data Fisher’s exact test used if appropriate. *P*-values ≤0.05 were considered as statistically significant and indicated in the figures as follows: *** *p* ≤ 0.001, ** *p* ≤ 0.01, * *p* ≤ 0.05.

For the calculation of metabolic half-life the following formula was used: $G(x)= Go\times {\left(\frac{1}{2}\right)}^{\frac{x}{t.}}$

(G(x) = serum concentration (24 h), G_0_ = serum concentration (0 h), x = 24 h, t = metabolic half-time)

## Results

### 2250 has a cytotoxic effect on all cell lines in a dose dependent manner

**Fig. 2:**
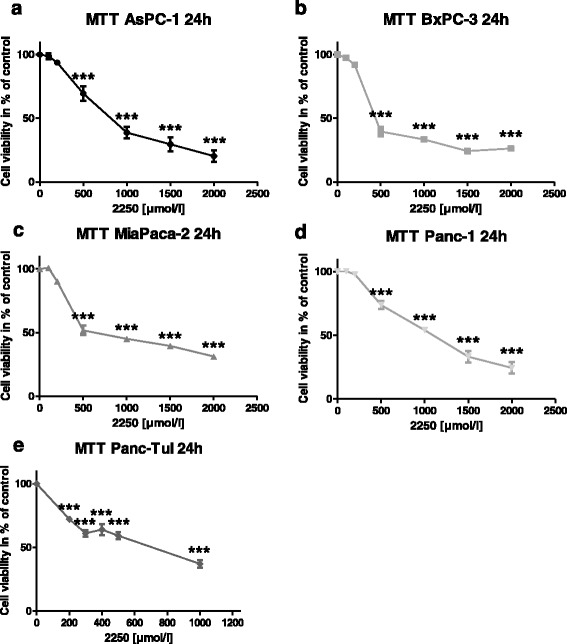
Effects of 2250 in different malignant cell lines measured by MTT-assay. AsPC-1 (**a**), BxPC-3 (**b**), MiaPaca-2 (**c**), Panc-1 (**d**) and Panc TuI cells (**e**) were incubated with 2250 (100, 200, 500, 1000, 1500, 2000 μmol/l) and ddH_2_O (control) for 24 h and submitted to a MTT-assay. Values are means ± SEM of 6 replicates of three independent experiments with consecutive passages. Asterisk symbols indicate differences between control, which was adjusted to 100% and 2250 treatment. *** *p* ≤ 0.001, ** *p* ≤ 0.01, * *p* ≤ 0.05, n.s. *p* > 0.05 (one-way ANOVA followed by Tukey’s post-hoc test)

To determine the effect of substance 2250 on the cell viability the OD (optical density), MTT tests were conducted. As indicated in Fig. 2, incubation with 2250 in increasing concentrations (100, 200, 500, 1000, 1500, 2000 μmol/l) for 24 h resulted in a dose dependent reduction of living cells - as measured by MTT assay. The substance 2250 in the cell line Panc-TuI was only analyzed up to a concentration of 1000 μmol/l, because this cell line was used additionally for in vivo treatment. The concentrations used in vitro were the important ones for in vivo treatment.

In all cell lines, even the lowest concentration of 500 μmol/l led to a significant reduction of cell viability ranging between 39.4% (± 3.8%) (BxPC-3) and 73.8% (± 3.3%) (Panc-1) which was significantly lower compared to untreated controls (100%) with ddH_2_O (Fig. 2). However, at the following concentration of 1000 μmol/l 2250, the monitored reduction of cell viability was more than 50% in four out of five cell lines. This pronounced reduction, mediated by 2250 in a concentration of 1000 μmol/l resulted in values of living cells between 33.3% (± 1.2%) for BxPC-3 and 59.1% (± 2.6%) for PancTuI (Fig. 2b, e). The maximum dose of 2000 μmol/l led to an intense cytotoxic effect in all cell lines. As a result, in three cell lines a proportional doses-effect curve and in BxPC-3 as well as in MiaPaca2 a sigmoid character was observed (Fig. 2). The effective doses (ED) 50 were varying between the different cell lines from 221 μmol/l (BxPC-3) to 1100 μmol/l (Panc-1) within an incubation time of 24 h.

### 2250 inhibits proliferation of all cell lines in a dose dependent manner

**Fig. 3:**
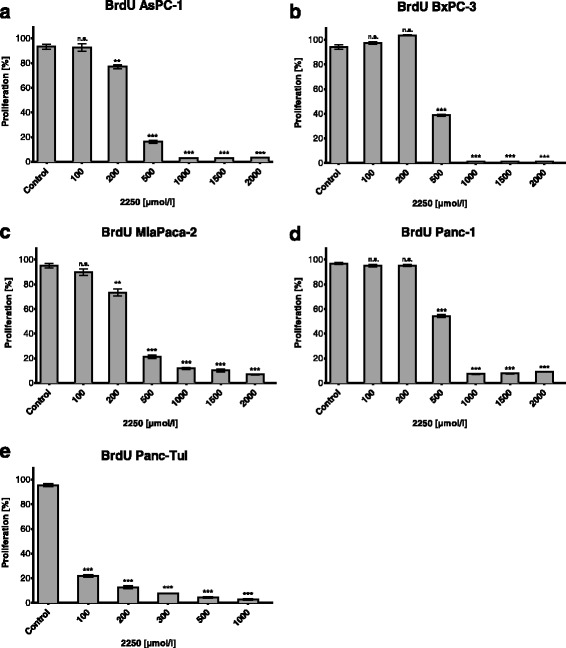
Effects of Taurolidine (TRD) on cell proliferation in different malignant cell lines measured by BrdU-assay. AsPC-1 (**a**), BxPC-3 (**b**), MiaPaca-2 (**c**), Panc-1 (**d**) and Panc TuI cells (**e**) were incubated with 2250 (100, 200, 500, 1000, 1500, 2000 μmol/l) and ddH_2_O (control) for 6 h and submitted to a BrdU-assay. Values are means ± SEM of 8 replicates of three independent experiments with consecutive passages. Asterisk symbols on columns indicate differences between control, which was adjusted to 100% and 2250 treatment. *** *p* ≤ 0.001, ** *p* ≤ 0.01, * *p* ≤ 0.05, n.s. *p* > 0.05 (one-way ANOVA followed by Tukey’s post-hoc test)

To examine the effect of substance 2250 on cell proliferation in a culture model, BrdU assays were conducted. As indicated in Fig. 3, incubation with substance 2250 in increasing concentrations (100, 200, 500, 1000, 1500, 2000 μmol/l) for 6 h resulted in a dose dependent reduction of proliferating cells. In three cell lines, even a concentration of 200 μmol/l 2250 was capable of inhibiting proliferation leading to values of proliferating cells ranging between 12.7% (± 1.3%) (Panc-TuI) and 77.2% (± 1.6%) (AsPC-1) which was significantly lower compared to untreated controls (100%) with ddH_2_O (Fig. 3). The concentration of 500 μmol/l 2250 significantly inhibited proliferation in all analyzed cell lines. This inhibition resulted in amounts of proliferating cells between 4.3% (± 0.6%) for Panc-TuI and 54.3% (± 1.2) for Panc-1 (Fig. 3 d, e). The maximum dose of 2000 μmol/l implicated an extended inhibition of proliferation in all cell lines, so the dose response for cell proliferation could be characterized as proportional in all five pancreatic cancer cell lines (Fig. 3).

Table 1 shows the relative amounts of cell viability and cell proliferation of three different cell lines upon treatment with substances TRLT and 2250 (2000 μmol/l each). In all analyzed cell lines substance 2250 shows a substantial higher impact on cell viability as well as on cell proliferation. In AsPC-1 and Panc-1 substance 2250 is even twice as effective as TRLT.

**Table 1:** Results of MTT and BrdU assay of substance 2250 compared with TRLT in three different cell lines

	AsPC-1		BxPC-3		Panc-1	
	TRLT	2250	TRLT	2250	TRLT	2250
MTT assay cell viability (%)	48.78 ± 2.8	21.00 ± 3.8	36.01 ± 7.3	26.24 ± 0.3	65.24 ± 1.5	24.4 ± 4.6
BrdU Assay cell proliferation (%)	8.5 ± 0.9	3.34 ± 0.2	6.66 ± 0.7	1.0 ± 0.2	16.32 ± 1.2	9.14 ± 0.3

### 2250 induces apoptotic cell death in all cell lines

**Fig. 4:**
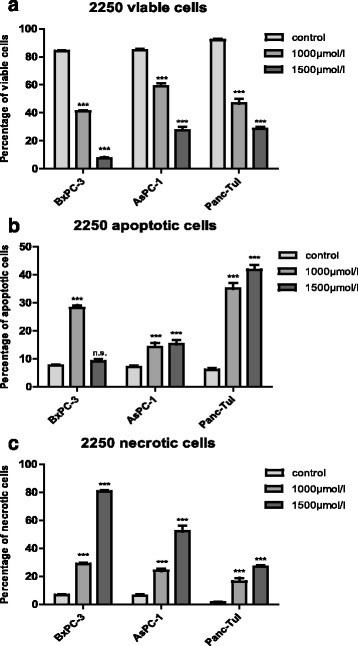
Effects of 1000 μmol/l and 1500 μmol/l 2250 on viability, apoptosis and necrosis in different malignant pancreatic cell lines measured by FACS analysis. AsPC-1 (**a**), BxPC-3 (**b**) and Panc-TuI (**c**) cells were incubated with 2250 (1000 and 1500 μmol/l) and ddH_2_O (control) for 24 h. The percentages of viable, apoptotic and necrotic cells were determined by FACS-analysis with Annexin V-FITC and Propidiumiodide. Values are means ± SEM of 4–6 independent experiments with three consecutive passages. Asterisk symbols on columns indicate differences between control and 2250 treatment. *** *p* ≤ 0.001, ** *p* ≤ 0.01, * *p* ≤ 0.05, n.s. *p* > 0.05 (one-way ANOVA followed by Tukey’s post-hoc test)

Additional FACS analysis revealed the impact of substance 2250 on apoptotic-, as well as on necrotic cell death. As summarized in Fig. 4, incubation of three cell lines for 24 h with substance 2250 with a concentration of 1000 μmol/l and 1500 μmol/l resulted in a significant reduction of viable cells compared to control treatment with ddH_2_O as evaluated by FACS analysis with Annexin V-FITC and PI. The significant reduction of cell viability by 1000 μmol/l 2250 was paralleled by a significant increase of apoptotic cells in all cell lines (Fig. 4). Cell viability following incubation with 1000 μmol/l was varying between 41.1% (±0.7%) for BxPC-3 and 59.0% (±1.9%) for AsPC-1 cells. The strong impact on cell viability was paralleled by a significant apoptotic effect in all cell lines ranging between 14.2% (± 1.5%) apoptotic cells for AsPC-1 and 35.0% (± 2.01%) for Panc-TuI. The contribution of necrosis to the loss of cell viability was smaller. However, a significant increase in necrotic cells between 16.4% (± 2.5%) for Panc-TuI and 28.9% (± 1.0%) for BxPC-3 was observed (Fig. 4).

The following incubation with 1500 μmol/l of 2250 showed a pronounced and significant reduction in cell viability between 28.5% (± 1.5%) for Panc-TuI and 7.6% (± 0.8%) for BxPC-3 cells. This reduction in cell viability was also paralleled by a significant increase of apoptotic cells – but only in two of three cell lines (AsPc-1, Panc-TuI). In BxPc-3 no significant apoptotic alteration was detected. However, BxPc-3 cells responded towards 1500 μmol/l with a strong necrotic effect 80.6% (± 1.1%) which was highest observed among all cell lines and concentrations (Fig. 4).

### The radical scavenger N-acetylcysteine (NAC) and pancaspase inhibitor z-VAD show divergent effects on 2250 induced cell death

To evaluate the contribution of Caspase or reactive oxygen species mediated cell death to the observed effects of substance 2250 co-incubation experiments with substance 2250 and NAC or z-VAD were performed.

**Fig. 5:**
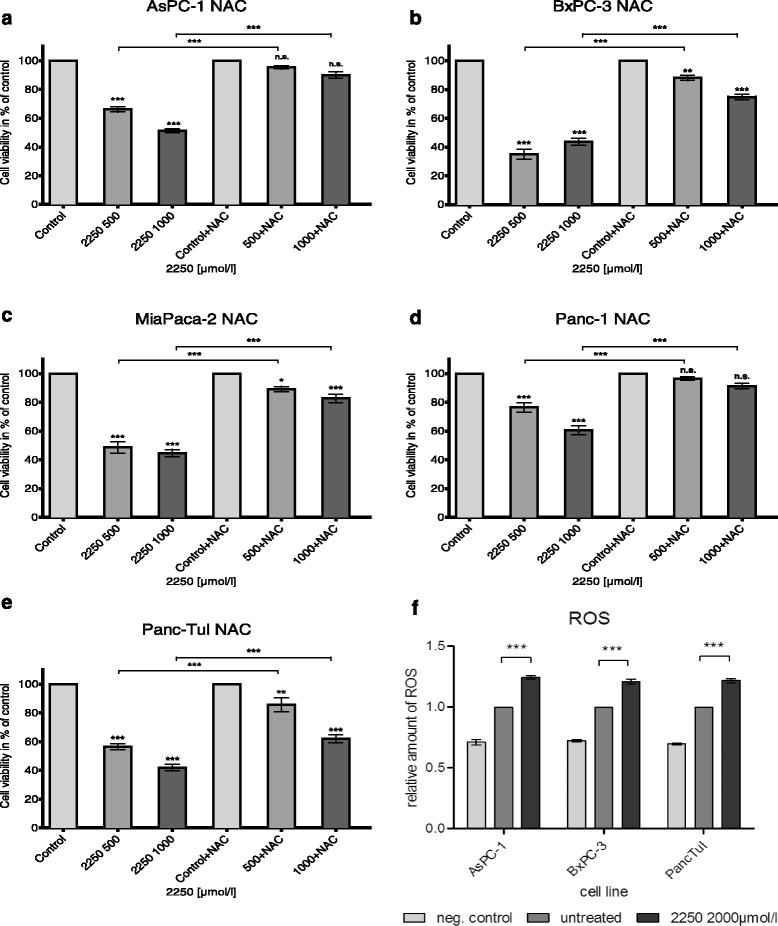
Effects of NAC on 2250 induced cell death in different malignant cell lines measured by MTT (**a**-**e**); impact of 2250 on the cellular level of ROS (f). AsPC-1 (**a**), BxPC-3 (**b**), MiaPaca-2 (**c**), Panc-1 (**d**) and Panc TuI (**e**) cells were incubated with either 2250 (500, 1000 μmol/l), NAC (5 mmol/l) or the combination of both agents (2250 500, 1000 μmol/l + NAC 5 mmol/l) and ddH_2_O as control for 24 h and submitted to a MTT-assay. The level of ROS was analyzed in untreated compared to 2250 treated cells, additional NAC treatment served as a neg. Control (**f**). Values are means ± SEM of 6 replicates of three independent experiments with consecutive passages. Asterisk symbols indicate differences between control, which was adjusted to 100% and 2250 treatment. *** *p* ≤ 0.001, ** *p* ≤ 0.01, * *p* ≤ 0.05, (one-way ANOVA followed by Tukey’s post-hoc test)

In AsPc-1 and Panc-1 cells, co-incubation of 2250 with NAC for 24 h led to a complete protection towards 2250 induced cell death. NAC entirely abrogated the 2250 induced reduction of viable cells leading to cell viability, equal to untreated controls (Fig. 5a, d). In BxPC-3, MiaPaca-2 and Panc-TuI, the co-incubation of 2250 with NAC was characterized by a strong protection of cell viability. However, there was no complete protection in the amount of viable cells compared to untreated controls. The effect could only be characterized as a partial protection (Fig. 5b, c, e).

**Fig. 6:**
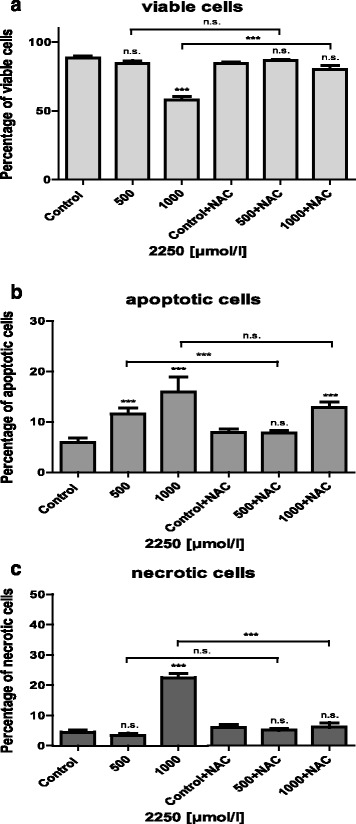
Effects of NAC on 2250 induced cell death in cell line AsPC-1 measured by FACS analysis. AsPC-1 cells were incubated with either 2250 (500, 1000 μmol/l), NAC (5 mmol/l) or the combination of both agents (2250 500, 1000 μmol/l + NAC 5 mmol/l) and ddH_2_O as control for 24 h. The percentage of viable (**a**), apoptotic (**b**) and necrotic (**c**) cells were determined by FACS analysis. Values are means ± SEM of 4–6 replicates of three independent experiments with consecutive passages. Asterisk symbols indicate differences between control, which was adjusted to 100% and 2250 treatment. *** *p* ≤ 0.001, ** *p* ≤ 0.01, * *p* ≤ 0.05, n.s. *p >* 0.05 (one-way ANOVA followed by Tukey’s post-hoc test)

Further data of FACS analysis of AsPC-1 co-incubated with 2250 and NAC for 24 h shown in Fig. 5 confirmed previous results. In contrast to the MTT assay, the FACS analysis shows this effect on the different cell populations more in detail. The co-incubation was characterized by a completely protection against the 2250 induced reduction of viable cells leading to a significant increase of viable cells (Fig. 6a). No differences from untreated controls were detected. Together with a small but significant reduction of apoptotic cells under treatment with a concentration of 500 μmol/l 2250, a complete reduction of necrotic cells could be achieved under treatment with 1000 μmol/l 2250 (Fig. 6b, c). Furthermore the cellular level of ROS is significantly increased in cells treated with substance 2250. Fig. 5f provides an exemplary presentation of tested cell lines. This effect can be reversed by additional treatment with NAC.

All pancreatic cancer cell lines which were analyzed did not show any detectable effect on cell viability after z-VAD co-incubation (data not shown).

### Detection of the maximal tolerable dose of substance 2250

By analyzing the acute toxicity, the highest concentration of 2000 mg/kg*BW was toxic in nude mice. The body weight was reduced about 1 g in 48 h and the mouse died 5 days after treatment. For the other concentrations no changes in body weight and vital function could be observed.

Determining the chronic toxicity it could be observed that concentrations higher than 1000 mg/kg*BW are toxic in nude mice. While treatment with 500 mg/kg*BW no changes in body weight and vital function could be monitored.

### Analysis of metabolic half-life in blood

**Fig. 7:**
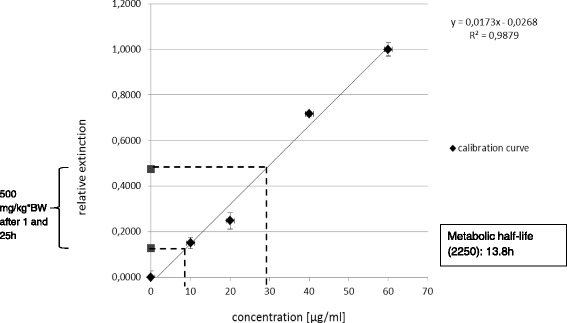
calibration curve of metabolic half-life measurement. The calibration curve consisting of five different concentrations of substance 2250 served as a standard for the calculation of the metabolic half-life of substance 2250 in nude mice, which resulted in a value of 13.8 h. The according measured parameters are shown in Table 2

For determining the metabolic half-life of the new substance 2250 in blood of nude mice a colorimetric assay using NASH reagent was used. The alignment of the measured values of substance 2250 in mice serum after 1 h and 25 h with the calibration curve yielded a metabolic half-life of 13.8 h (Fig. 7, Table 2).

**Table 2:** Measured parameters of metabolic half-life calculation

Time of blood sampling	Relative extinction	Concentration in serum [μg/ml]
1 h	0.475	29.044
25 h	0.128	8.954

### 2250 induces reduction of subcutaneous tumor growth in vivo

Finally, to elucidate the effect of substance 2250 in vivo, we tested the impact of the treatment of xenografts derived from cancer cell lines and patient tissue in different tumor mouse models.

**Fig. 8:**
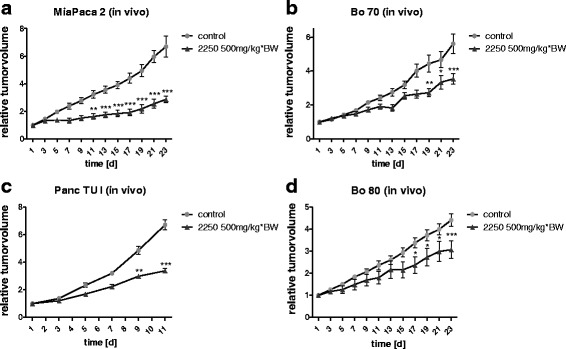
Effects of 500 mg/kg*BW 2250 on the subcutaneous tumor growth in nude mice in vivo. Nude mice with tumors of MiaPaca-2 (**a**), Panc-TuI (**c**) or patient tissue (**b**, **d**) were incubated with 2250 (500 mg/kg*BW) and ddH_2_O (control) for up to 3 weeks. The tumor volume was measured on alternating days for up to 3 weeks. Asterisk symbols indicate differences between control and 2250 treatment. *** *p* ≤ 0.001, ** *p* ≤ 0.01, * *p* ≤ 0.05, n.s. *p* >0.05 (one-way ANOVA followed by Tukey’s post-hoc test)

As indicated in Fig. 8, the intraperitoneal (ip) application of 2250 (500 mg/kg*BW) reduced the subcutaneous tumor growth in vivo in tumors induced by established pancreatic cancer cell lines (MiaPaca-2, Panc-TuI) as well as in xenograft models from patient tissue. The ip application of 2250 led to a significant reduction of 57% of relative tumor volume in comparison to the control group after an application period of 3 weeks in tumors caused by the established cell line MiaPaca-2 (Fig. 8a). In a second tumor model evoked by the established cell line Panc-TuI (Fig. 8b) almost the same inhibition of tumor growth was observed by ip application of 2250 (500 mg/kg*BW). A significant reduction of 49.7% of relative tumor growth was detected after an application period of 2 weeks. The tumor volume endpoint of 1000 mm^3^ was reached after this treatment period and the experiment was terminated. The subcutaneous tumor, derived from patient tissue (Bo 70) was analyzed in a separate tumor model in which a significant reduction of tumor growth of 36.8% under the treatment with substance 2250 could be observed (Fig. 8c). In a second patient derived xenograft (Bo 80) almost the same inhibition of approximately 30% could be detected (Fig. 8d).

## Discussion

The anti-neoplastic effects of the recently developed, novel substance 2250 have not been published yet. During development of the compound, a cytotoxicity test was performed in the glioma cell line LN 229 which indicated a promising anti-neoplastic capacity of the substance 2250 (EC 50 = 55 μg/ml after 24 h) [unpublished data], similar in potency to TRD. The anti-neoplastic activity of the parent compound TRD has been demonstrated in many studies on different cancer entities in vitro as well as in vivo [18, 20, 21] . Even pilot clinical studies confirmed previous results [16, 18, 22]. Therefore, the aim of this study was to analyze anti-neoplastic effects of substance 2250 in comparison to its structural analogue TRLT, the main derivative of its effective parent compound TRD.

In the first part of this study we determined dose-response characteristics and analyzed the relative contribution of apoptosis and necrosis during substance 2250 induced cell death. In all five different malignant pancreatic cancer cell lines the dose response effects of substance 2250 were nearly homogenous. We found a pattern which is characterized by a *proportional or sigmoidal dose effect* where increasing concentrations of substance 2250 led to an increase of cell death after 24 and 48 h. This pattern could be observed for the cytotoxicity of the substance via MTT assay, as well as for the anti-proliferative effect by BrdU analysis. This *proportional dose effect* pattern, observed during this study, is analog to those described in several previous studies for the treatment with TRD [23–26].

Three pancreatic cancer cell lines (AsPC-1, BxPC-3 and PancTuI) also displayed a dose-response relationship concerning the relative distribution of viable, apoptotic and necrotic cells determined by FACS analysis, where the amount of viable cells decreased with increasing concentration of substance 2250. The incubation with 1000 μmol/l of substance 2250 was characterized by apoptotic as well as necrotic cell death whereby the apoptotic effect was the predominant characteristic. In contrast, the incubation with 1500 μmol/l of substance 2250 was characterized by a pronounced necrotic effect in BxPC-3. This effect became more obvious in AsPC-1. Opposite to this, in PancTuI cells treated with 1500 μmol/l the amount of apoptotic cells prevailed. This dose depending ratio of viable, apoptotic and necrotic cells is according to previous studies with TRD in our group [15] and by others [13, 26]. Regarding the pronounced necrotic effect of rising concentrations of substance 2250, only speculations are possible. Whereby, cell culture models could reveal a potential explanation. Generally, apoptosis is due to caspase-dependent or independent cell destruction and to phagocytosis of the apoptotic cells. In a cell culture setting, which lacks phagocytosis a secondary necrosis follows, which is characterized by the same appearance of primary necrosis [27].

In our in vitro studies there is no possibility of phagocytosis by inflammatory cells, because they are not present in our cell culture model. The marker, used for necrosis (PI) could ultimately not differentiate between a substance-induced primary necrosis and a secondary necrosis - due to the lack of phagocytosis. Another possible explanation might be the induction of a programmed necrosis by substance 2250 itself, as shown previously for Taurolidine [26]. Programmed necrosis is, besides apoptosis and autophagy, the third type of programmed cell death, which involves cell swelling, organelle dysfunction and cell lysis. Programmed necrosis can be induced by different signaling pathways a. o. via activation of death receptors or cell stress can induce activation of receptor- interacting-protein kinases RIP1 and RIP3, which influence mitochondria to induce ROS increase [27, 28]. Further work is required to clarify whether substance 2250 lead to the induction of programmed necrosis on such a high level, at least in BxPC-3 and AsPC-1 at high concentrations.

The second part of this study deals with the contribution of reactive oxygen species (ROS) to substance 2250 induced cell death and the analysis of another major cell death associated pathway, the caspase pathway. Previous studies with TRD revealed an involvement of ROS 12, 40 [29], triggering apoptosis via caspase-independent pathways [12, 20], caspase-dependent pathways 40 [15, 20, 25] as well as necroptosis and autophagy [26]. Furthermore, cell death, induced by ROS, has been shown to be prevented by applying the radical scavenger N-acetycysteine (NAC) [12, 25, 29]. Hence, in this study both the cellular ROS induction upon treatment and co-incubation experiments with either the radical scavenger NAC for inhibition of oxidative stress or the pan caspase inhibitor z-VAD for involvement of the caspase pathway were performed. Substance 2250 clearly induced ROS production in all tested cell lines. This effect could be completely canceled by the radical scavenger NAC. In line with this result, all analyzed pancreatic cancer cell lines responded to NAC co-incubation with an attenuated cell death, induced by substance 2250. Nevertheless, the extent of protection from cell death was divergent between the analyzed cell lines, ranging from partial protection (BxPC-3, MiaPaca-2, PancTuI) to complete protection (AsPC-1, Panc-1). In general, ROS is regarded as being an ambiguous compound in terms of anti-neoplastic activity [30]. On the one hand, it is able to support tumor cell proliferation and survival [14, 30]. On the other hand, excessive ROS generation in tumor cells can induce cell death. This therapeutic effect is used by many chemotherapeutics as platinum, arsenic, ascorbate or piperlongumine [13, 30–32]. In conclusion, the generation of ROS and the consequent activation of following pathways is an obvious explanation of the induction of cell death by substance 2250 in pancreatic cancer cell lines. Thus, ROS induced cell death may not be the universal mechanism of substance 2250, but our experiments highlight its central role in programmed cell death (PCD).

We also analyzed another major cell death associated pathway mentioned in the literature referring TRD induced cell death - the caspase pathway [20, 25, 33]. Among the literature, the activation of the caspase pathway by TRD has been mentioned for several cell lines, but the findings were divergent between different tumor entities. Furthermore, caspase independent PCD induced by TRD was reported in the literature as well [12, 20]. The analyzed pancreatic cancer cell lines (AsPC-1, BxPC-3) were not protected via inhibition of pan-caspases from PCD induced by substance 2250 in our experiments. Previous studies of our group determined that pancreatic cancer cell lines were not protected from PCD caused by TRD, via inhibition of pan caspases, either [15]. The response observed in pancreatic cancer cell lines regarding the inhibition of 2250 induced cell death, via the pan-caspase inhibitor zVAD, leads to the assumption, that especially for pancreatic cancer cell lines the caspase dependent PCD plays only a minor role. Therefore, other pathways like necroptosis or caspase independent programmed cell death may play a more considerable role. These pathways are well discussed in the latest literature related to cancer therapies [34–37]. Further studies in this field are necessary to clarify the involvement of caspase independent types of programmed cell death following the treatment with the substance 2250.

On the basis of this foregone promising in vitro results*,* we analyzed the anti-neoplastic effects of substance 2250 in vivo. Prior to the in vivo studies the metabolic half-life of the new substance 2250 was determined, due to the fact that Taurolidine shows a very short metabolic half-life of only 1–2 h in healthy human volunteers [22]. Our analysis yielded a metabolic half-life of substance 2250 of 13.8 h in nude mice, displaying a much higher metabolic stability compared to TRD. Based on the fact that mice have a higher metabolism than humans, it can be assumed, that the metabolic half-life of substance 2250 in humans is even higher. TRD was previously found to suppress tumor growth of different tumor entities in rodents [10, 11, 20, 21, 38–45]. The ip application of TRD inhibits the ip tumor growth of colonic carcinoma [42], mesothelioma [31], malignant melanoma [13, 45] and pancreatic carcinoma [43, 44]. It could also be shown that the intravenous (iv) application of TRD decreases the ip tumor growth as well [38, 40, 41, 46]. Existing studies also have experiences with the effect of TRD application on subcutaneous tumor growth. In colon carcinoma the subcutaneous tumor growth was not influenced by TRD application [38, 40], whereas the subcutaneous tumor growth in malignant melanoma [13, 47] and prostate carcinoma [48] was inhibited by TRD instillation ip and iv.

Therefore our experimental in vivo study was based on the hypothesis that substance 2250 could also inhibit the growth of pancreatic cancer after ip administration in an established cell line model and a xenograft model with patient tissue in nude mice. We observed a significant reduction of subcutaneous tumor growth in tumors induced by established pancreatic cancer cell lines (MiaPaca 2, PancTuI) as well as in xenograft models from patient tissue (Bo70, Bo80). In tumors caused by established pancreatic cell lines, the ip application led to a significant reduction by approximately 50% of relative tumor volume in comparison to the controls. These results were confirmed by the xenograft models derived from patient tissue, where a reduction of relative subcutaneous tumor volume by 30–40% was observed after treatment with substance 2250.

## Conclusion

In conclusion, this is the first study providing an evaluation of substance 2250 induced cell death among several pancreatic cancer cell lines in vitro and inhibition of pancreatic tumor growth in vivo*.* Substance 2250 is characterized by a clear dose response relationship due to the fact that all analyzed cells lines were susceptible to substance 2250 induced cell death. Functional analysis of the involvement of ROS driven and caspase activated PCD showed, that ROS plays a fundamental role inducing cell death in pancreatic carcinoma. In contrast, PCD by caspase activation was less important. Furthermore substance 2250 seems to effectively inhibit pancreatic cancer tumor growth in mice, simultaneously displaying a higher metabolic stability, which will be a strong benefit towards clinical practice. These encouraging results are the basis for further functional analysis and initial clinical studies for this promising agent. It remains to be seen whether 2250, in analogy to TRD, also exerts anti-inflammatory activity by reducing pro-inflammatory cytokines.

## Abbreviations

Annexin V-FITC: Annexin V-Fluorescein
BrdU: 5-bromo-2-deoxyuridine
DMEM: Dulbecco’s modified eagle medium
DMSO: Dimethylsulfoxide
ELISA: Enzyme Linked Immunosorbent Assay
FACS: Fluoreszcence-activated cell scanning
Ip: Intraperitoneal
NAC: N-acetlycysteine
OD: Optical density
PCD: Programmed cell death
PDAC: Pancreatic ductal adenocarcinoma
PI: Propidiumiodide
ROS: Reactive oxygen species
RPMI 1640: Roswell park memorial institute medium 1640
TRD: Taurolidine
TRLT: Taurultam
UICC: Union international contre le cancer
z-VAD: carbobenzoxy-valyl-alanyl-aspartyl-[O- methyl]- fluoromethylketone
